# Unravelling the functional development of vertebrate pathways controlling gaze

**DOI:** 10.3389/fcell.2023.1298486

**Published:** 2023-10-26

**Authors:** Marta Barandela, Carmen Núñez-González, Daichi G. Suzuki, Cecilia Jiménez-López, Manuel A. Pombal, Juan Pérez-Fernández

**Affiliations:** ^1^ CINBIO, Universidade de Vigo, Neurocircuits Group, Campus universitario Lagoas, Marcosende, Vigo, Spain; ^2^ Faculty of Life and Environmental Sciences, University of Tsukuba, Tsukuba, Japan; ^3^ Department of Functional Biology and Health Sciences, Facultade de Bioloxía-IBIV, Universidade de Vigo, Campus universitario Lagoas, Marcosende, Vigo, Spain

**Keywords:** visual system, eye movements, vestibulo-ocular reflex, optokinetic reflex, pretectum, optic tectum, goal-oriented movements, lamprey

## Abstract

Animals constantly redirect their gaze away or towards relevant targets and, besides these goal-oriented responses, stabilizing movements clamp the visual scene avoiding image blurring. The vestibulo-ocular (VOR) and the optokinetic reflexes are the main contributors to gaze stabilization, whereas the optic tectum integrates multisensory information and generates orienting/evasive gaze movements in all vertebrates. Lampreys show a unique stepwise development of the visual system whose understanding provides important insights into the evolution and development of vertebrate vision. Although the developmental emergence of the visual components, and the retinofugal pathways have been described, the functional development of the visual system and the development of the downstream pathways controlling gaze are still unknown. Here, we show that VOR followed by light-evoked eye movements are the first to appear already in larvae, despite their burrowed lifestyle. However, the circuits controlling goal-oriented responses emerge later, in larvae in non-parasitic lampreys but during late metamorphosis in parasitic lampreys. The appearance of stabilizing responses earlier than goal-oriented in the lamprey development shows a stepwise transition from simpler to more complex visual systems, offering a unique opportunity to isolate the functioning of their underlying circuits.

## 1 Introduction

Ocular movements have gradually emerged across evolution depending on new motor and perceptual needs. The oldest eye movements appeared with the aim of immobilizing the visual image on the retina: the vestibulo-ocular (VOR) and the optokinetic (OKR) reflexes (Land, 2015). These reflexes are conserved throughout evolution and their appearance dates back in the phylogenetic tree of vertebrates to the position of lampreys, belonging to the oldest group of living vertebrates (Rovainen, 1976). The interface for the VOR lies in the vestibular nuclei, divided in lampreys in the anterior (AON), intermediate (ION) and posterior (PON) octavomotor nuclei, which send vestibular information to the three motor nuclei that control the extraocular muscles: oculomotor (nIII), trochlear (nIV) and abducens (nVI; Pombal et al., 1996; Fritzsch, 1998). As in other vertebrates, the pretectum (PT) generates OKR, sending visual information to the oculomotor nuclei both directly and through the vestibular nuclei (Giolli et al., 2006; Masseck and Hoffmann, 2009; Wibble et al., 2022). Thus, the circuits controlling these reflexes are very similar to other vertebrates meaning that they appeared at the dawn of vertebrate evolution and are largely conserved.

Lampreys also possess the main centers involved in goal-oriented gaze redirection. As in mammals, the optic tectum (OT; mammalian superior colliculus) has a layered structure and receives visual information retinotopically, aligned with other sensory modalities (de Arriba Mdel and Pombal, 2007; Jones et al., 2009; Cornide-Petronio et al., 2011; Kardamakis et al., 2016). Sensory information is integrated by the tectal circuits that prioritize salient stimuli and encode the appropriate behavioral response by generating orienting/evasive movements (Saitoh et al., 2007; Kardamakis et al., 2015; Kardamakis et al., 2016; Kardamakis et al., 2017; Suzuki et al., 2019). The OT also receives other inputs that can modulate its motor commands, including those from the basal ganglia and cortex (Stephenson-Jones et al., 2011; Pérez-Fernández et al., 2014; Pérez-Fernández et al., 2017; Pérez-Fernández et al., 2021; Ocaña et al., 2015). Additionally, a visual area is present in the lamprey pallium, suggesting that a primordial blueprint of the visual cortex is present in these animals (Suryanarayana et al., 2020; Suryanarayana et al., 2021), and therefore all the main visuomotor areas are present in lampreys.

The visual system of lampreys shows a unique development, suggested to reflect its evolution, because of their complex life cycle (Suzuki and Grillner, 2018). Larvae are photophobic, filter feeders, and their eyes are eyespots covered with skin, not being functional to form images (Kleerekoper, 1972). After a very long larval period (five to 7 years; Hardisty, 2006), lampreys undergo a metamorphosis in which some species develop structures for their new parasitic life, including functional eyes (Kennedy and Rubinson, 1977; Suzuki and Grillner, 2018). The structures and circuitry necessary to use the visual system for advanced behaviors develop stepwise during the larval period, and the visual system completes its development during metamorphosis (Kennedy and Rubinson, 1977; Suzuki and Grillner, 2018). In early larvae (<60–70 mm), retinopretectal connections are already established, and direct retinal projections to the nucleus of the medial longitudinal fascicle (nMLF) generate a simple circuit possibly involved in negative phototaxis (de Miguel et al., 1990; Cornide-Petronio et al., 2011; Suzuki et al., 2015a; Suzuki et al., 2015b). At this stage, the OT shows an immature state and has no input from the retina, which is formed in larger larvae (de Miguel et al., 1990; Cornide-Petronio et al., 2011). The OT completes its development during metamorphosis. The extraocular muscles also develop gradually, starting before the development of the retinotectal projection (Suzuki et al., 2016), and the oculomotor, trochlear and abducens neurons that innervate those muscles also develop gradually, differentiating earlier than the extraocular muscles (Fritzsch et al., 1990; Pombal et al., 1994; Suzuki and Grillner, 2018). Retinal development is also gradual. Prolarvae and early larvae exhibit a primary retina without horizontal and amacrine cells, essential for image-forming vision (Villar-Cerviño et al., 2006), and during the larval period the retina expands and undergoes extensive cell differentiation completing its development during metamorphosis (Kennedy and Rubinson, 1977). During this period, the eye also acquires a chambered structure with a spheric lens, and the overlying skin becomes transparent (Dickson and Collard, 1979). Regarding stabilizing gaze movements, the circuits mediating VOR are established during the larval period (Pombal et al., 1996; Fritzsch, 1998), although whether this reflex is functional at this stage is unknown. Regarding OKR, no data exist in larva.

The large degree of similarities between the visual system of lampreys and that of other vertebrates, together with its gradual development through a very long period, offer a unique window to isolate functional developmental aspects of visuomotor behaviors and their underlying circuits (Suzuki and Grillner, 2018). Although the order of appearance of the different visual components, and the development of the retinofugal pathways have been described (Kennedy and Rubinson, 1977; de Miguel et al., 1990; Cornide-Petronio et al., 2011), their functionality is unknown. Moreover, although some of the motor pathways that generate orienting, evasive, and eye movements are well described in adults (Saitoh et al., 2007; Kardamakis et al., 2015; Kardamakis et al., 2016; Kardamakis et al., 2017; Suzuki et al., 2019), their development is still unknown. In this study, we show that larval lampreys have coordinated eye movements in the form of VOR, and that eye movements can also be evoked by light stimuli. However, the direct pretectal and tectal motor outputs to reticulospinal neurons are mostly established during metamorphosis, although these structures can trigger motor responses before, which are conveyed through polysynaptic pathways. Interestingly, tectal and pretectal projections are established earlier in non-parasitic lampreys. Given the high degree of conservation of the analyzed circuits, our results provide insights to understand the functional development of the vertebrate visual system.

## 2 Results

### 2.1 Larval lampreys exhibit coordinated eye movements

**FIGURE 1:**
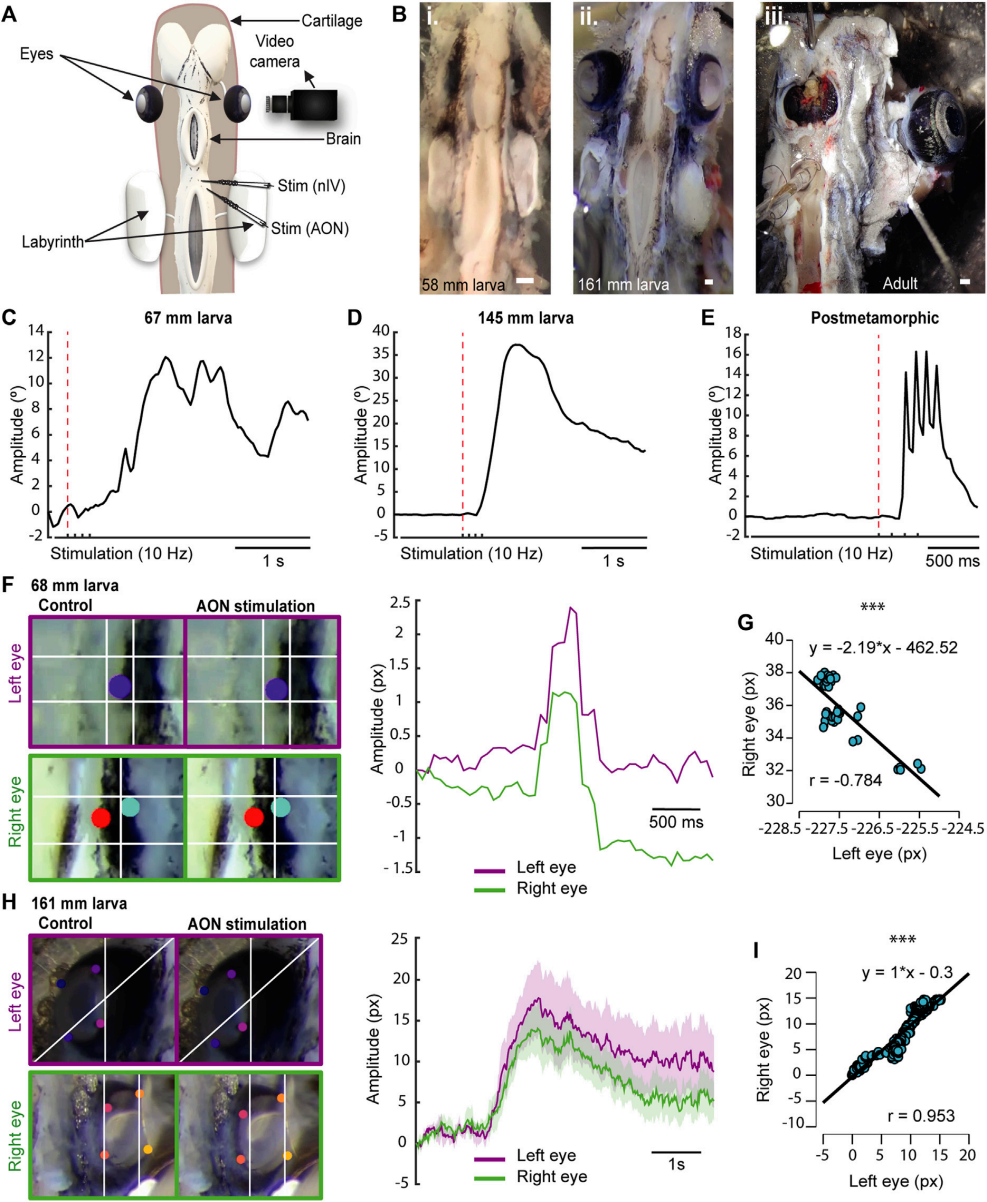
Lamprey larvae exhibit eye movements. **(A)** Schematic showing the preparation used to monitor eye movements. **(B)** Preparations of 58 (Bi) and 161 (Bii) mm long larvae showing eye development in relation to body size. A preparation of an adult animal is shown in Biii. **(C–E)** Traces representing the eye position in response to a four pulses stimulation (10 Hz) of the anterior octavomotor nucleus (AON) in a 67 **(C)** and a 145 **(D)** mm larva, and a postmetamorphic **(E)** lamprey. The red dotted line indicates when the first pulse was applied. **(F)** Right: Traces showing the position of the right (green) and left (purple) eyes in response to a four pulses 10 Hz AON stimulation in a 68 mm larva, showing coordinated movements. Note that the trajectory of the right eye is inverted to better reflect the coordination between both eyes (see also Movie 3). Left: Frames showing the position of the eyes (top, left eye; bottom, right eye) before (left) and after (right) AON stimulation. **(G)** Graph showing the correlation between the movements of both eyes of the 68 mm larva shown in **(F)**, indicating their coordination after AON stimulation. **(H)** Right: Traces showing the position of the right (green) and left (purple) eyes in response to a four pulses 10 Hz AON stimulation in a 161 mm larva. Data are shown as mean ± s.d. Left: Frames showing the position of the eyes (top, left eye; bottom, right eye) before (left) and after (right) AON stimulation. **(I)** Graph showing the correlation between the right and left eye movements of the 161 mm larva shown in **(H)**. Abbreviations: nIV Trochlear Motor Nucleus. Scale bar = 300 µm in **(Bi-iii)**.

Given that the eye muscles already develop during the larval period (∼60 mm length), and the cranial nerve supply for these muscles (i.e., the oculomotor, trochlear, and abducens nerves) appears even earlier (Fritzsch et al., 1990; Pombal et al., 1994; Suzuki et al., 2016), we first explored whether this motor infrastructure is already functional in lamprey larvae despite their burrowed lifestyle and underdeveloped eyes. We used a preparation exposing the eyes and brain, so that we could stimulate different areas and monitor eye movements via video recordings and/or electromyograms (EMGs) in the extraocular muscles (Figure 1A). The progressive development of the eyes can be seen in the preparations from larvae of different sizes and adults (Figure 1Bi-iii).

To investigate the presence of eye movements, we first used video tracking (N = 31, one eye was analyzed per animal) analyzing the videos with DeepLabCut (Mathis et al., 2018). A video camera was placed facing one of the eyes, and either the AON or the nIV were electrically stimulated (Figure 1A; 4 pulses, 10 Hz). The first question was whether larvae have eye movements, and we thus started with large larvae (>140 mm, N = 6). Reliable eye movements were evoked as shown in the 145 mm larva of Figure 1D (Movie 1). We then investigated smaller larvae, observing eye movements in larvae as small as 67 mm (Figure 1C; N = 4). Although electric stimulation activated in many cases antagonistic muscles resulting in eye movements that were not purely rotational but accompanied by a translational movement of the eye towards the cartilage, thus hampering a comparative analysis of eye movement amplitudes, we observed that the range of movement varied depending on the individual’s development, with larger larvae exhibiting larger eye movement amplitudes than smaller ones (Figures 1C,D). Interestingly, in larvae, a single slow movement was observed after applying four stimulation pulses (Figures 1C,D; Movie 1), whereas late metamorphic (N = 4), postmetamorphic (N = 2), and adult (N = 1) lampreys exhibit four rapid movements, one for each stimulation pulse (Figure 1E; Movie 2). These differences in eye movement velocity could be due to a low degree of development of the oculomotor circuits and/or the extraocular muscles, or rather to a mechanical constrain of the eye given the underdevelopment of the orbit. To investigate this, electromyograms in the dorsal rectus (DR) muscle of larvae (N = 3) were performed in response to electrical stimulation of the AON or the nIV. In Supplementary Figure S1 (top trace), the EMG activity in the DR of an adult lamprey (N = 1; see also Rovainen, 1976) in response to a four pulses electric stimulation of the AON (10 Hz) is shown, and activity can be seen in response to each of the pulses. Below, the activity in the DR of a 161 mm larva is shown. Although the signal recorded was weaker due to the small size of the extraocular muscle, a clear response was observed after each of the four stimulation pulses. This indicates that the muscle contracts in a similar fashion to adults, and that the differences in eye movement kinematics are due to the underdevelopment of the eye/orbit.

We next investigated whether eye movements in larvae are coordinated as in adults (Wibble et al., 2022), or if there are movements preceding this coordination. For this, we placed a video camera on top of the animal to track both eyes in response to AON stimulation. Coordinated movements occur as soon as the eyes move (Movie 3). In Figures 1F,G (Pearson’s correlation analysis; r = −0.784; *p* < 0.001; Movie 3), the coordinated trajectories of the eyes in response to AON stimulation (4 pulses, 10 Hz) are shown in a 68 mm larva. This coordination was observed in all recorded larvae >67 mm (N = 23), although the correlation was higher in pre-metamorphic larvae (Figures 1H,I; Pearson’s correlation analysis; r = 0.953; *p* < 0.001; Movie 4). These results demonstrate that, despite living borrowed and having underdeveloped eyes covered with skin, larvae have coordinated eye movements as soon as their extraocular muscles and their innervation (Pombal et al., 1994) develop.

### 2.2 VOR is the first type of eye movement that emerges

**FIGURE 2:**
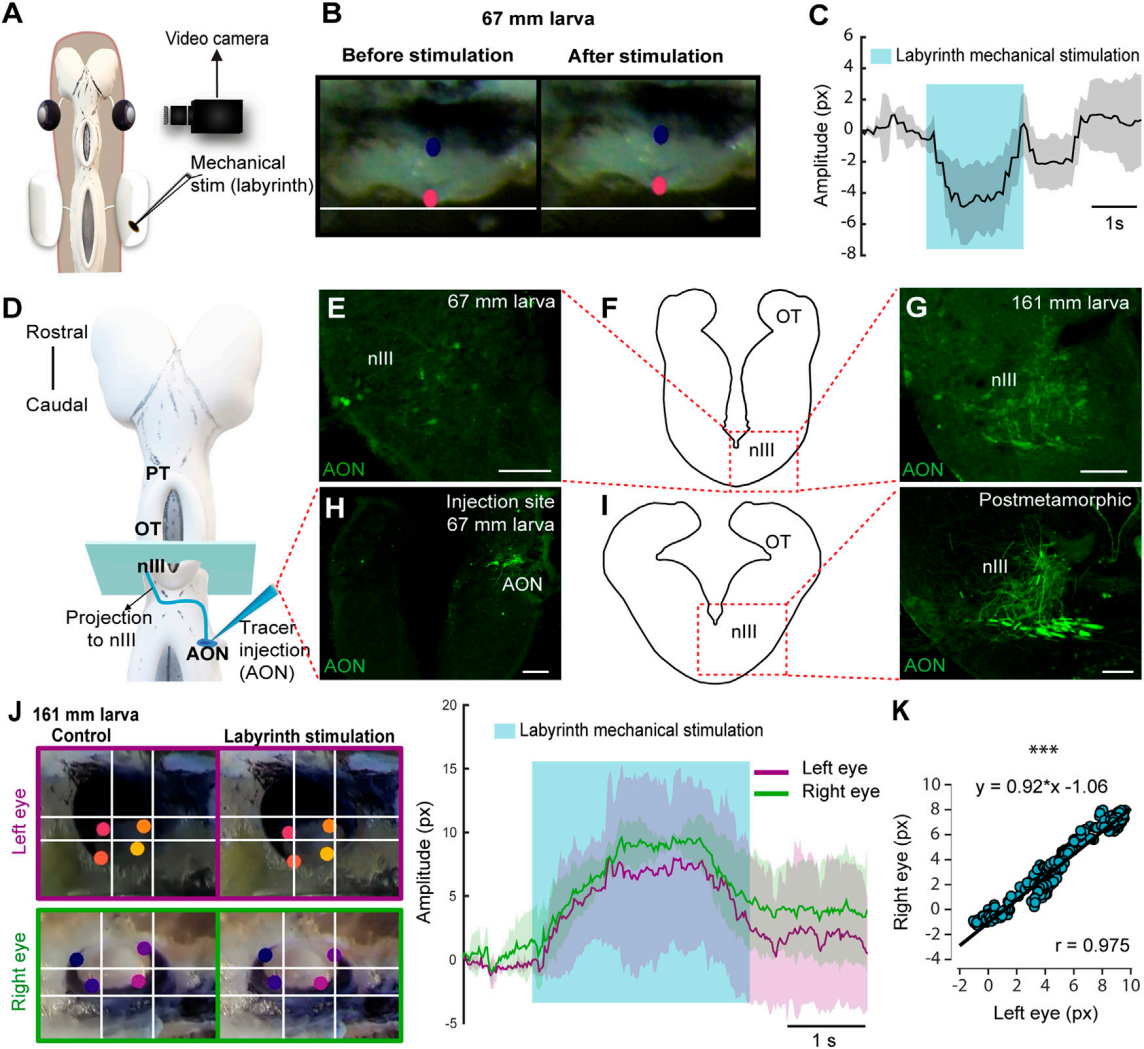
Lamprey larvae exhibit VOR. **(A)** Schematic showing the preparation used to monitor eye movements in response to labyrinth mechanical stimulation. **(B)** Eye of a 67 mm larva before (left) and after (right) vestibular stimulation. **(C)** Graph showing the eye movement of the same larva after labyrinth stimulation. The shaded area indicates the duration of the vestibular stimulation. **(D)** Schematic dorsal view of a larval brain showing the location of the tracer injection site (Anterior octavomotor nucleus, AON) and its anterograde projections (blue line) reaching the contralateral oculomotor nucleus (nIII). **(E–I)** The level of the drawings shown in **(F)** and **(I)** (left) corresponds to the blue rectangle in **(D)** and the dotted rectangle in both drawings indicates the location of the photomicrographs at the level of the contralateral nIII showing axons anterogradely labeled from the AON in a 67 **(E)** and a 161 **(G)** mm larva and a postmetamorphic animal [**(I)** right]. **(H)** Representative injection site in the AON of a 67 mm larva. **(J)** Left: Frames showing eye position (top, left eye; bottom, right eye) before (left) and after (right) vestibular stimulation. Right: Traces showing the position of the right (green) and left (purple) eyes in response to labyrinth stimulation in a 161 mm larva. The blue shaded area denotes the duration of the vestibular stimulation. **(K)** Graph showing the correlation between the movements of both eyes. Data are shown as mean ± s.d. Abbreviations: OT Optic Tectum, PT Pretectum. Scale bar = 100 µm in **(E)** and **(H)**; 50 µm in **(G)** and **(I)**.

The VOR probably emerged as the first gaze stabilization reflex, would therefore be evolutionarily the earliest, and the first to appear in development (Straka and Baker, 2013). In lamprey larvae, vestibular information from the AON is initially directed to the spinal cord (SC) when they are 27–30 mm long. In larvae >60 mm, the AON axons project ventrorostrally and eventually form synapses with the nIII (Pombal et al., 1994; Pombal et al., 1996; Fritzsch, 1998). Interestingly, this was the smallest size at which we could detect eye movements, and no eye movements were evoked stimulating the nIII, nIV or nVI nerves in smaller animals that did not respond to AON stimulation. This suggests that the VOR is the first eye movement that appears in lampreys. Thus, we decided to analyze the VOR nature of the first eye movements in ∼60 mm larvae. For this, we first confirmed that eye movements elicited by AON stimulation are the result of activating the vestibular system by monitoring eye movements in response to mechanical stimulation of the labyrinth in larvae >60 mm (Figure 2A; N = 3). This resulted in eye movements (Figures 2B,C; Movie 5), like those evoked by AON stimulation (see above), confirming their VOR nature.

To test if the VOR becomes functional as soon as the underlying neural circuits appear, tracer injections were performed in the AON (N = 12) to determine the larval size at which the VOR circuit develops (Figures 2D–I). Tracer injections confirmed the projections to the nIII previously shown both in larvae and adults (Pombal et al., 1996; Fritzsch, 1998; Wibble et al., 2022). Anterogradely labeled fibers were found in the nIII of >60 mm larvae (Figures 2E–H; N = 6) that also showed eye movements in response to AON stimulation, but no anterogradely labeled fibers were found in smaller larvae (<60 mm, N = 4; not shown). The AON projection to the nIII increased considerably in larger larvae, postmetamorphic (N = 3) and adult lampreys (N = 3; Figures 2F,G,I, see also Wibble et al., 2022). The increase in fiber number throughout development was also observed at their crossing point, caudal to the nIII (Supplementary Figure S2A,B,C). Next, we tested whether the coordinated eye movements in response to AON stimulation could also be evoked in response to labyrinth stimulation, confirming their VOR nature (N = 2). Figures 2J,K illustrates a 161 mm larva that shows significant correlation between the movement of both eyes (Pearson’s correlation analysis; r = 0.975; *p* < 0.001). These results show that VOR is the first type of eye movements that emerges.

### 2.3 Lamprey larvae exhibit eye movements in response to light

**FIGURE 3:**
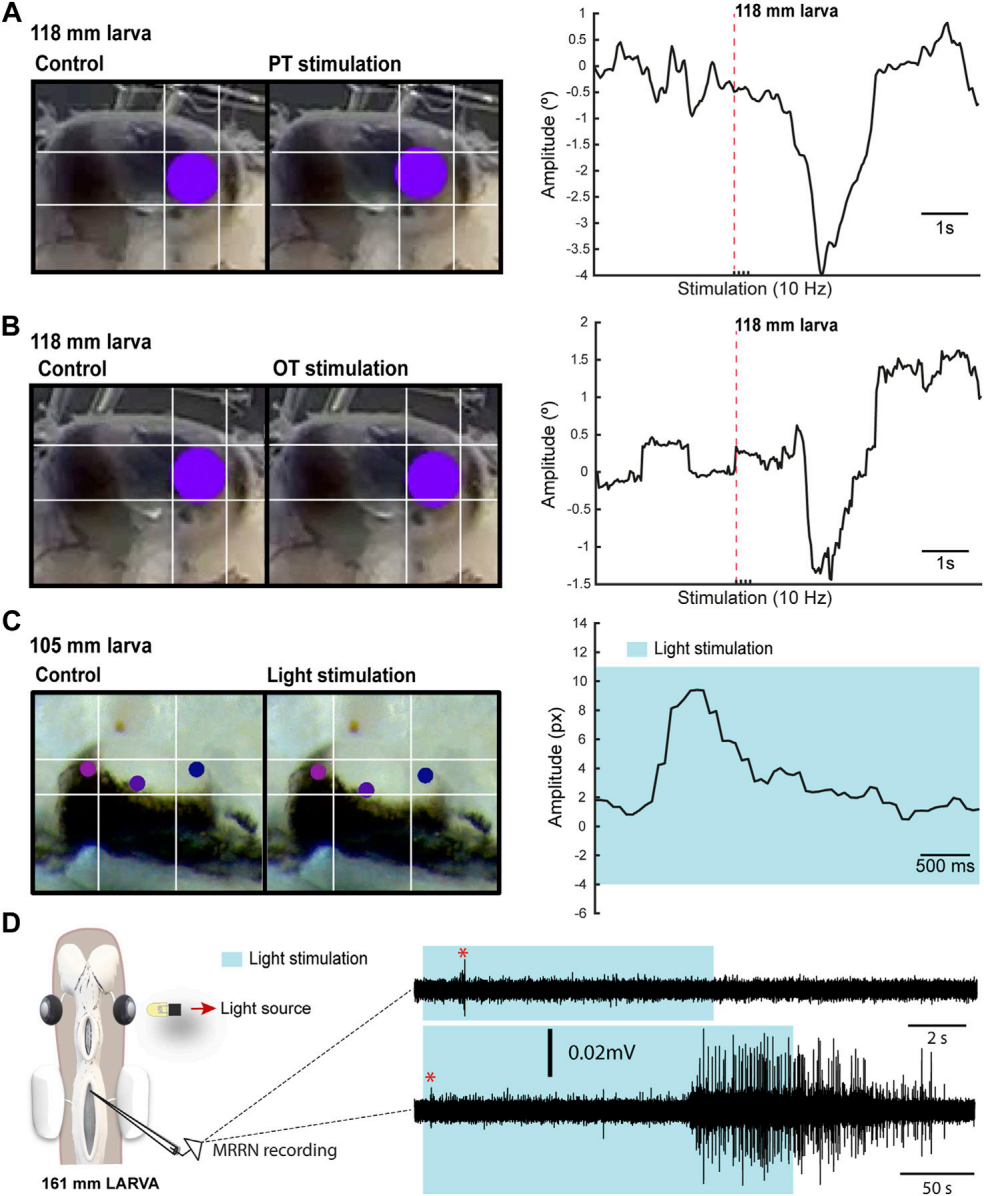
Light-evoked responses. **(A)** Left: Eye of a 118 mm larva before (left) and after (right) a four pulses electric stimulation of the pretectum (PT; 10 Hz). Right: Graph showing the eye movement in response to the stimulation. In **(B)** the eye movement is shown in response to optic tectum (OT; 10 Hz) stimulation. The red dotted line in **(A)** and **(B)** indicates when the first pulse was applied. **(C)** Representative frames (left) showing the eye position before and after light stimulation for the eye movement shown in the graph (right). The shadowed area indicates light stimulation to both eyes. **(D)** Representative traces showing extracellular activity in the middle rhombencephalic reticulospinal nucleus (MRRN) of a 161 mm larva in response to a 10 s (top trace) and to a 50 s (bottom trace) light stimulation presented to one eye (see schematic). The shadowed area denotes the duration of the stimulation, whereas the red asterisk signals the short latency evoked responses.

The presence of VOR in larvae raised the question of whether other types of eye movements also appear during this period. In lampreys, visual information can generate eye movements mostly through three visual centers: the PT that mediates the optokinetic reflex and has been also suggested to mediate phototactic responses, the OT thought to mediate goal-oriented movements, and pallial regions thought to be the precursor of cortical regions (see above). Thus, we first performed electric stimulation of these areas to check whether eye movements could be evoked (N = 26). Eye movements were observed in larvae as small as 100 mm in response to both pretectal (N = 7; Figure 3A) and tectal stimulation (N = 5; Figure 3B). Interestingly, no eye movements could be evoked in these larvae stimulating the pallial visual area (Supplementary Figure S2D–E), nor activity could be evoked in the middle rhombencephalic reticulospinal nucleus (MRRN, Supplementary Figure S2F), and the same happened in metamorphic and recent postmetamorphic animals, although activity in the MRRN and/or eye movements were observed after tectal/pretectal stimulation (see below), in agreement with the late development of tectal and pretectal motor outputs (see below).

The presence of eye movements evoked from retinorecipient areas after electric stimulation raised the question of whether visual stimuli could also trigger eye movements. The low degree of retinal development indicates that larvae can only integrate basic visual features, and we therefore analyzed eye movements in response to light (N = 11). Although no eye movements were observed with light stimuli presented to one eye, these were evoked in response to broad light stimulation to both eyes in larvae >100 mm (Figure 3C; N = 5). We also tested whether extracellular activity could be light evoked in the MRRN, indicative of phototactic responses (see below). Weak short latency activity was observed in larvae >100 mm in response to light stimulation presented with a LED to one eye (Figure 3D; N = 2). Furthermore, a strong activation was evoked when light stimulation was maintained several seconds (Figure 3D, bottom trace). These results indicate that visual information can generate eye and body movements already in larvae, likely as basic phototactic responses mediated by the eyes. However, a contribution of deep brain photoreceptors cannot be totally excluded (Barreiro-Iglesias et al., 2017), although this is unlikely given that they are thought to regulate circadian and reproductive responses to light (Nakane et al., 2014). The presence of light-evoked eye movements suggested that OKR might be also present in larvae. To investigate this, we did tracer injections in the PT (N = 28) to see whether anterogradely labeled fibers were present in the nIII. However, no evidence of a PT to nIII connection was found (not shown), indicating that the anatomical substrate of OKR (Wibble et al., 2022) is missing at this stage.

### 2.4 Development of tectal and pretectal projections to the MRRN in parasitic lampreys

In adult lampreys, neurons in the deep layer of the OT send direct projections to the brainstem, contacting reticulospinal neurons in the MRRN. Tectal projections are both contra- and ipsilateral and control orienting and evasive responses, respectively (Kardamakis et al., 2015; Kardamakis et al., 2016; Kardamakis et al., 2017; Suzuki et al., 2019). The PT also sends direct projections to the reticular formation, providing an additional substrate for the visually evoked motor responses (Zompa and Dubuc, 1996; Ullén et al., 1997; Zompa and Dubuc, 1998; Capantini et al., 2017). However, when these circuits develop is not yet known. Thus, we combined extracellular recordings in the MRRN in response to OT and PT stimulation at different developmental stages with tracer injections in this region to uncover when these connections arise. Given that the stepwise development of the lamprey visual system is consequence of their very long larval period, we speculated that differences in the visual circuits controlling orienting and evasive responses might be present in lampreys with different lifestyles. Thus, we compared the tectal/pretectal outputs of a parasitic lamprey (*Petromyzon marinus*), with those of the non-parasitic Northern Japanese brook lamprey (*Lethenteron* sp. N).

**FIGURE 4:**
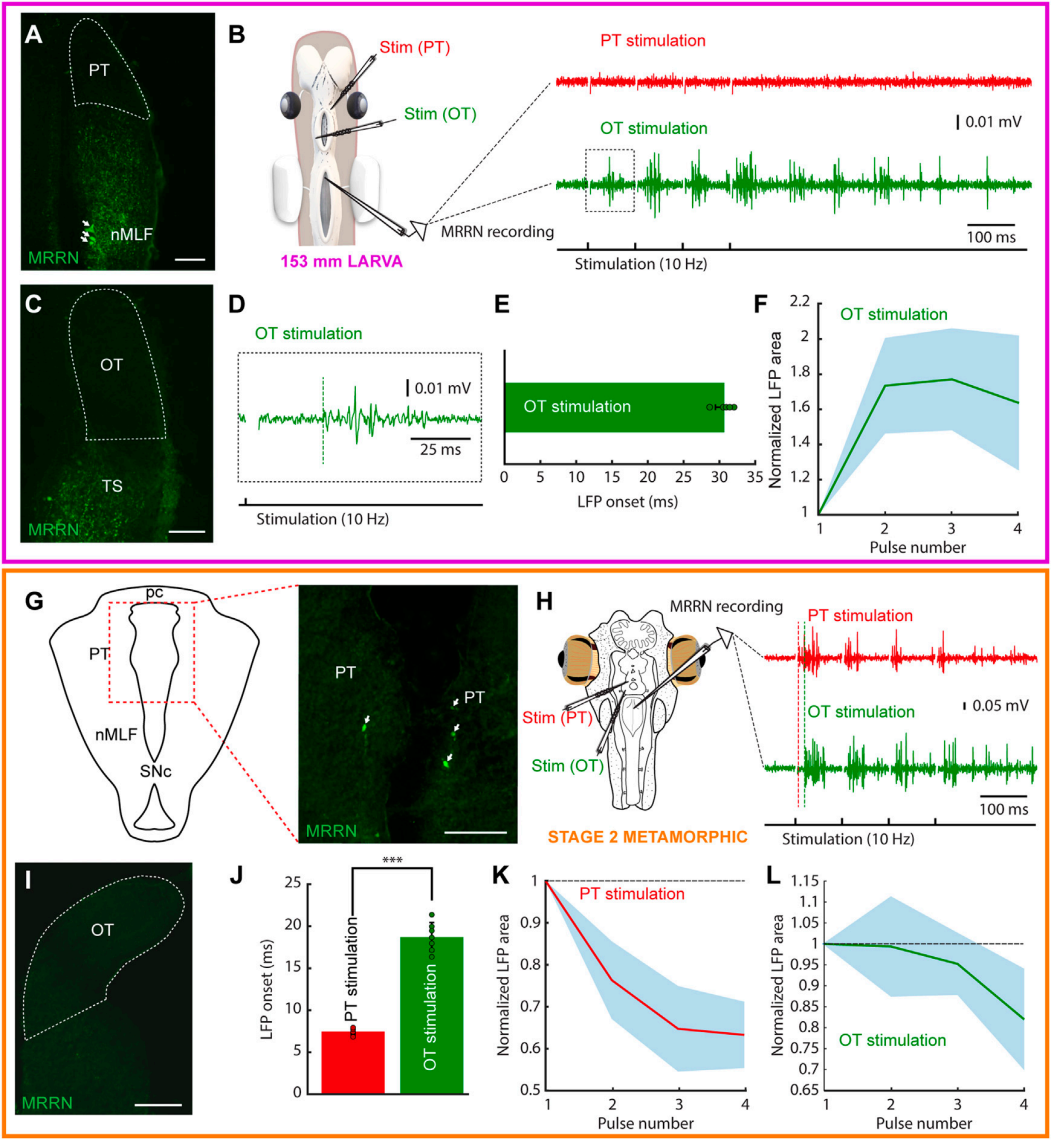
Tectal and pretectal motor outputs in larvae/early metamorphic. The colored rectangles group results belonging to the developmental stage indicated under the schematic of the experimental preparation (magenta: 153 mm larva; orange: stage 2 metamorphic). **(A)** Photomicrograph showing the lack of retrogradely labeled neurons in the pretectum (PT, indicated by a dashed line) after a dextran injection in the middle rhombencephalic reticulospinal nucleus (MRRN) of a 153 mm larva. Retrogradely labeled neurons can be seen in the nucleus of the medial longitudinal fasciculus (nMLF; arrows). **(B)** Schematic showing the preparation used to record neuronal activity in the MRRN of the same larva (left). PT stimulation did not evoke activity in the MRRN (right, red trace), whereas optic tectum (OT) stimulation resulted in extracellular activity (right, green trace). **(C)** No retrogradely labeled neurons were observed in the OT after a dextran injection in the MRRN (surrounded by a dashed line). **(D)** Magnified view of the region indicated by a dashed line square in the trace shown in **(B)** indicating the onset of the evoked response (vertical dashed green line). **(E)** Plot showing the average onset times of the responses evoked in the MRRN after OT stimulation. **(F)** Mean responses in the MRRN in response to 4 pulses OT stimulation (10 Hz), combining data of 5 larvae from 65 to 161 mm. **(G)** Schematic (left) indicating the location of the photomicrograph (right) showing bilateral retrogradely labeled MRRN projecting neurons in the periventricular aspect of the PT in a stage 2 metamorphic lamprey (arrows). **(H)** Left, schematic showing the preparation used to record extracellular activity in the MRRN (right) in response to PT (red trace) and OT (green trace) stimulation. Vertical dashed lines indicate the onset of the evoked responses. **(I)** Photomicrograph showing the lack of retrogradely labeled neurons in the OT after tracer injection in the MRRN. **(J)** Graph showing the difference between response onsets in the MRRN after PT (red) and OT (green) stimulation. **(K,L)** Graphs showing the mean MRRN activity in a stage 2 animal evoked by PT **(K)** and OT **(L)** stimulation. Values are normalized to the first local field potential (LFP). Stimulation artifacts were removed for clarity. Data are shown as mean ± s.d. Abbreviations: pc Posterior commissure, SNc Substantia Nigra pars compacta, TS Torus Semicircularis. Scale bar = 100 µm in **(A)**, **(C)**, **(G)** and **(I)**.

In parasitic lampreys, no evidence of projections from either OT or PT to the MRRN was found in larvae. Neurobiotin injections in the MRRN were performed (N = 12) and in all cases the tectal and pretectal regions were devoid of retrogradely labeled neurons (Figures 4A,C). In agreement with this, no activity was evoked in the MRRN after PT stimulation (Figure 4B, red trace). Interestingly, in a couple of larvae short latency responses were evoked in the MRRN in response to high intensity stimulation pulses in the PT (not shown), most likely due to the activation of nMLF dendrites shown to reach this area (Suzuki et al., 2015a; Suzuki et al., 2015b). Instead, responses were evoked in the MRRN of larvae >60 mm after tectal stimulation although only with long latency onsets, indicative of a polysynaptic pathway (Figures 4B,D, green trace, E: response onset = 30.68 ± 1.29 ms; n = 5). These responses were initially potentiating, but usually decayed after the third pulse (Figure 4F).

Given the lack of tectal and pretectal projections to the MRRN in larvae of parasitic lampreys, we next analyzed their presence in metamorphic animals (N = 7). Tracer injections in the MRRN resulted in retrogradely labeled neurons in the PT already in the earliest metamorphic stage that we could analyze (stage two, Youson and Potter, 1979; Supplementary Figure S3A–C). However, only some retrogradely neurons were found in the periventricular region (Figure 4G). Still, clear activity could be recorded in the MRRN after PT stimulation (Figure 4H, red trace; 10 Hz, four pulses). Noticeably, anterogradely fibers were observed at this and later stages (N = 6) in the nIII after tracer injections in the PT, showing that the OKR substrate also develops early during metamorphosis (not shown). However, no retrogradely labeled neurons were found in the OT after tracer injection in the MRRN (Figure 4I). The electrophysiological activity recorded in the MRRN after pretectal stimulation showed a short latency (7.43 ± 0.37 ms), significantly shorter than that evoked after tectal stimulation (18.64 ± 1.71 ms; Figure 4J; unpaired *t*-test, *p* < 0.0001; n = 14), thus confirming the direct pretectal projections to the MRRN and the lack of monosynaptic tectal projections.

**FIGURE 5:**
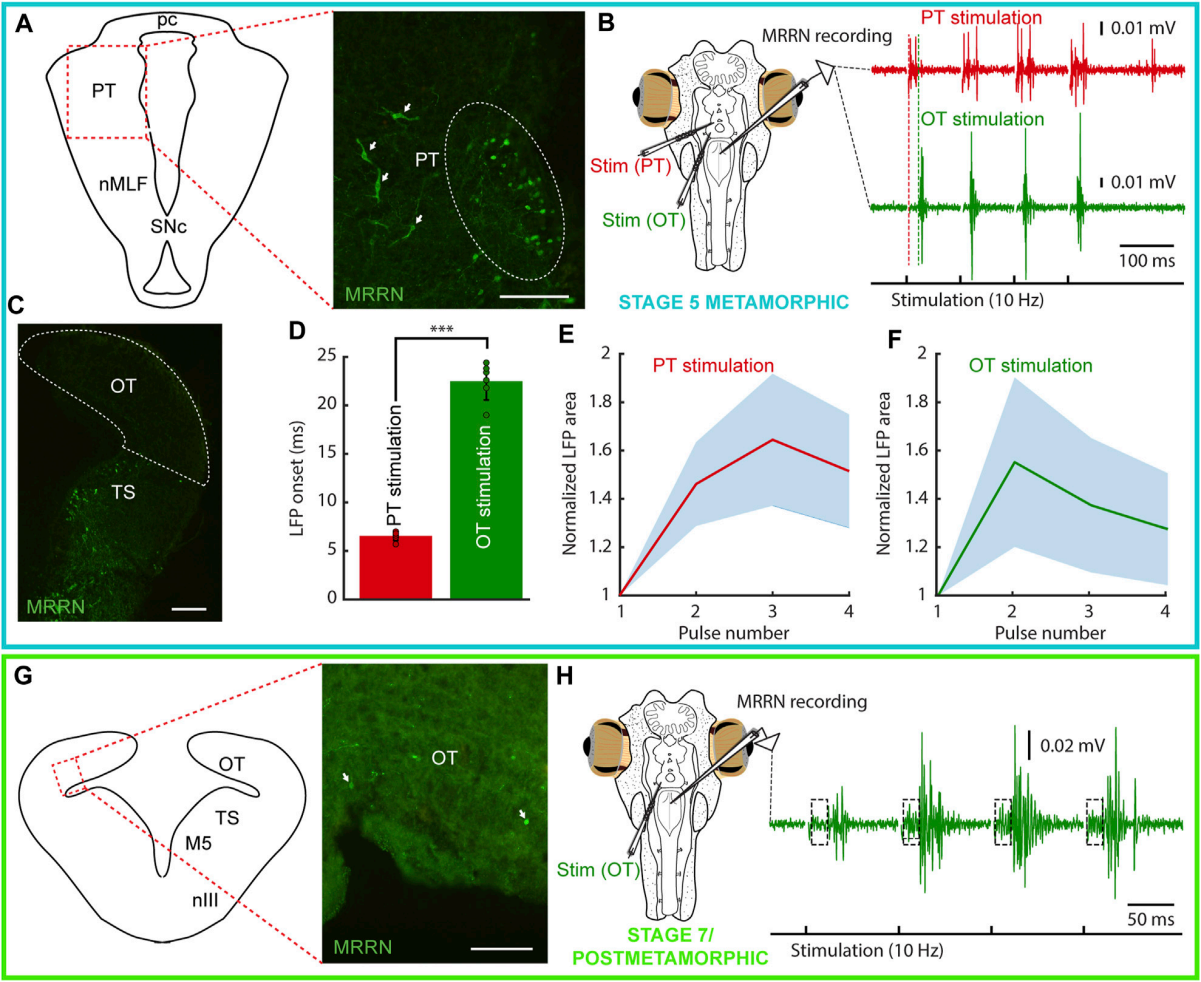
Tectal and pretectal projections to the MRRN in metamorphic and postmetamorphic lampreys. The colored rectangles group results belonging to the developmental stage indicated under the schematic of the experimental preparation (blue: stage 5 metamorphic; green: stage 7 metamorphic/postmetamorphic). **(A)** Schematic (left) indicating the location of the photomicrograph (right) showing retrogradely labeled neurons in the pretectum (PT) of a stage 5 metamorphic lamprey after a Neurobiotin injection in the middle rhombencephalic reticulospinal nucleus (MRRN). Projection neurons can be observed both in the periventricular region (dashed line oval), and in lateral aspects (arrows). **(B)** Extracellular responses in the MRRN after PT (red trace) and optic tectum (OT, green trace) stimulation. The onset of the extracellular activity is indicated by a dashed red line for PT stimulation, and a dashed green line for OT stimulation. **(C)** Photomicrograph showing that no retrogradely labeled neurons can be seen in the OT (dashed area) of a stage 5 metamorphic lamprey after Neurobiotin injection in the MRRN. **(D)** Graph showing that the onsets of MRRN responses evoked by PT stimulation (red) were significantly shorter than those evoked by OT stimulation (green). **(E, F)** Graphs showing the mean responses evoked in the MRRN of a stage 5 metamorphic animal evoked by PT **(E)** and OT **(F)** stimulation in response to 4 pulses (10 Hz). Values are normalized to the first local field potential (LFP). **(G)** Schematic (left) indicating the location of the photomicrograph (right) showing a few retrogradely labeled neurons from the MRRN (arrows) in the OT of a late metamorphic animal. **(H)** Extracellular responses in the MRRN of a late metamorphic animal in response to OT stimulation (4 pulses, 10 Hz). A two-components response can be observed: a fast onset weak response (indicated by a dashed line rectangle) followed by a stronger signal. In the electrophysiological traces, stimulation artifacts were removed for clarity. Data are shown as mean ± s.d. Abbreviations: nMLF Nucleus of the Medial Longitudinal Fasciculus, pc Posterior commissure, SNc Substantia Nigra pars compacta, TS Torus Semicircularis, nIII Oculomotor Nucleus, M5 Retinopetal Nucleus of Schöber. Scale bar = 100 µm in **(A, G)**; 200 µm in **(C)**.

In later metamorphic stages (stage five and later), tracer injections in the MRRN resulted in retrogradely labeled neurons both in periventricular and lateral portions of the PT, like in adults (Figure 5A; Zompa and Dubuc, 1996; El Manira et al., 1997; Capantini et al., 2017). The conspicuous population of fusiform neurons extending their dendrites into the optic tract reported in adults could also be observed at this stage (Figure 5A, arrows). Extracellular recordings in the MRRN in response to pretectal stimulation resulted in short latency responses (6.55 ± 0.46 ms; Figure 5B, red trace), in agreement with a monosynaptic input (von Twickel et al., 2019). In the OT, no retrogradely labeled neurons were found after tracer injection in the MRRN (Figure 5C, dashed area). Accordingly, although extracellular activity could be consistently evoked in the MRRN in response to tectal stimulation (Figure 5B, green trace), these responses had a long latency (22.5 ± 1.94 ms), significantly longer than the pretectal evoked responses (unpaired *t*-test, *p* < 0.0001; Figure 5D, see vertical dashed lines, and 5D; n = 12), indicative of a polysynaptic pathway similar to larvae (see above). Interestingly, only in late metamorphic/early postmetamorphic lampreys retrogradely labeled neurons could be detected in the OT, and still in small numbers (Figure 5G). Accordingly, weak responses with short latencies were observed in the MRRN after tectal stimulation, followed by a big response component, which corresponds to the polysynaptic response observed in previous stages (Figure 5H). Contrary to larvae, in metamorphic animals the MRRN responses after tectal or pretectal stimulation were not consistently potentiating, and both inhibitory (Figures 4K,L) and potentiating cases (Figures 5E,F) were found. The presence of polysynaptic connections in larvae suggests that visual stimuli integrated by the OT and the PT can evoke motor responses through the reticular formation, most likely related to phototactic responses, as suggested by the above presented experiments using light stimulation. However, in parasitic lampreys, the full map of projections is not completely developed until very late stages of the metamorphosis, with the beginning of their parasitic and migratory feeding life.

### 2.5 Earlier development of tectal projections in non-parasitic lampreys

**FIGURE 6:**
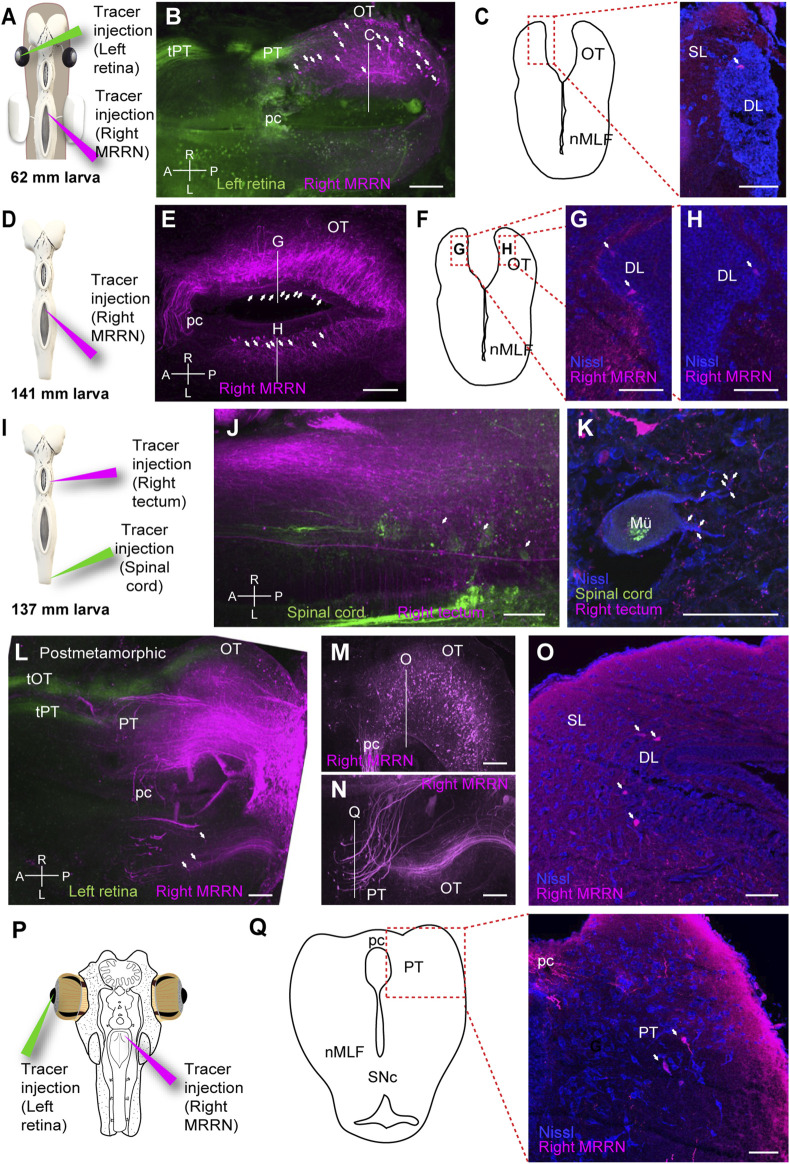
Tectal and pretectal projections to the MRRN in non-parasitic lampreys. **(A)** Schematic indicating the location of the tracer injections sites in the left retina and the right middle rhombencephalic reticulospinal nucleus (MRRN). **(B)** Whole-brain confocal image showing dual labeling of the optic nerve fibers (green) and MRRN projecting tectal neurons (magenta) in a 62 mm larva, dorsal view. The optic nerve predominantly projects to the pretectum (PT) via the pretectal tract (tPT). The optic tectum (OT) contains retrogradely labeled neurons in its rostrocaudal extent (arrows). **(C)** Schematic (left) indicating the location of the photomicrograph (right) at the level depicted in B showing a retrogradely labeled neuron in the OT of a 62 mm larva. The labeled neuron is not in the superficial (SL) but in the deep periventricular (DL, arrow) layer. **(D)** Schematic indicating the location of the tracer injection site in the MRRN. **(E)** Whole-brain confocal image showing MRRN-projecting tectal neurons (magenta) in a 141 mm larva, dorsal view (arrows). **(F–H)** Schematic and photomicrographs at the levels depicted in **(E)** showing retrogradely labeled neurons in the left **(G)** and right **(H)** DL of the OT in a 141 mm larva (arrows). The location of the photomicrographs in **(G)** and **(H)** is shown in the schematic in **(F). (I)** Schematic indicating the location of the tracer injection sites in the right OT and the spinal cord (SC). **(J)** Whole-brain confocal image showing dual labeling of retrogradely labeled SC-projecting cells (green) and anterogradely labeled tectal fibers (magenta) in a 137 mm larva, dorsal view. Numerous MRRN-projecting tectal fibers are observed (arrows). **(K)** Photomicrograph showing anterogradely labeled tectal terminals on a large Müller neuron (Mü) in the MRRN region. **(L)** Whole-brain confocal image showing dual labeling of the optic nerve fibers (green) and MRRN-projecting tectal neurons (magenta) in postmetamorphic animals, dorsal view. In addition to tPT, the tectal tract (tOT) of the optic nerve is observed. Some contralateral MRRN-projecting tectal neurons are also distinguished (arrows). **(M, N)** Region-specific images for the right **(M)** and left **(N)** OT, generated by different Z-stack projections to visualize ipsilateral MRRN-projecting tectal neurons and contralateral MRRN-projecting pretectal neurons, respectively. **(O)** Photomicrograph at the level depicted in **(M)** showing retrogradely labeled neurons from MRRN in the DL (arrows) of the ipsilateral OT. **(P)** Schematic indicating the location of the tracer injection sites in the left retina and the right MRRN. **(Q)** Schematic (left) indicating the location of the photomicrograph (right) at the level depicted in **(N)**, showing retrogradely labeled neurons from MRRN in the contralateral PT (arrows) in postmetamorphic animals. Abbreviations: A Anterior, L left, pc Posterior Commisure, nMLF Nucleus of the Medial Longitudinal Fasciculus, P posterior, R right, SNc Substantia Nigra pars compacta. Scale bars = 100 µm in **(B)**, **(E)**, **(J)**, **(L)**, **(M)**, and **(N)**; 50 µm in **(C)**, **(G)**, **(H)**, **(K)**, **(O)**, and **(Q)**.

The late appearance of the tectal and pretectal motor projections in parasitic lampreys indicates that the development of the visual system is completed in parallel with the biological needs of the animal. This raised the question of whether tectal and pretectal motor outputs develop differently in non-parasitic lampreys. Thus, we investigated tectal and pretectal projections in a non-parasitic, landlocked species (Northern Japanese brook lamprey, *Lethenteron* sp. N). Whole-brain confocal imaging allowed us to confirm that the optic nerve fibers did not project to the OT but predominantly project to the PT in larvae <70 mm (Figures 6A,B; de Miguel et al., 1990; Cornide-Petronio et al., 2011). Nevertheless, retrograde labeling in these small larvae already showed both tectal and pretectal neurons projecting to the ipsilateral MRRN (N = 3/3; Figures 6A–C). In larvae >70 mm, contralaterally-projecting tectal neurons were also found in some specimens (N = 2/5; Figures 6D–H). Also, tracer injections into the OT (Figure 6I) resulted in anterogradely labeled fibers in the MRRN region in both large larvae (Figure 6J; N = 3) and postmetamorphic animals (not shown; N = 4). The labeled fibers in larvae were in close contact with the MRRN Müller neurons (Figure 6K), indicating that the tectal neurons are connected directly to the MRRN. Lastly, in postmetamorphic animals, both ipsi- and contralaterally projecting tectal neurons were observed (N = 4/4; Figures 6L–N,P). These labeled neurons were all in the deep layer (DL) of the OT (Figures 6C,G,H,O). Additionally, pretectal neurons projecting to the MRRN were also labeled on both ipsi- and contralateral sides in postmetamorphic animals (N = 4/4; Figures 6O,Q). These results show that the differentiation of the tectal projections to the MRRN is accelerated in the non-parasitic, landlocked lampreys compared to parasitic species.

### 2.6 The nMLF mediates the first tectal-evoked motor responses

**FIGURE 7:**
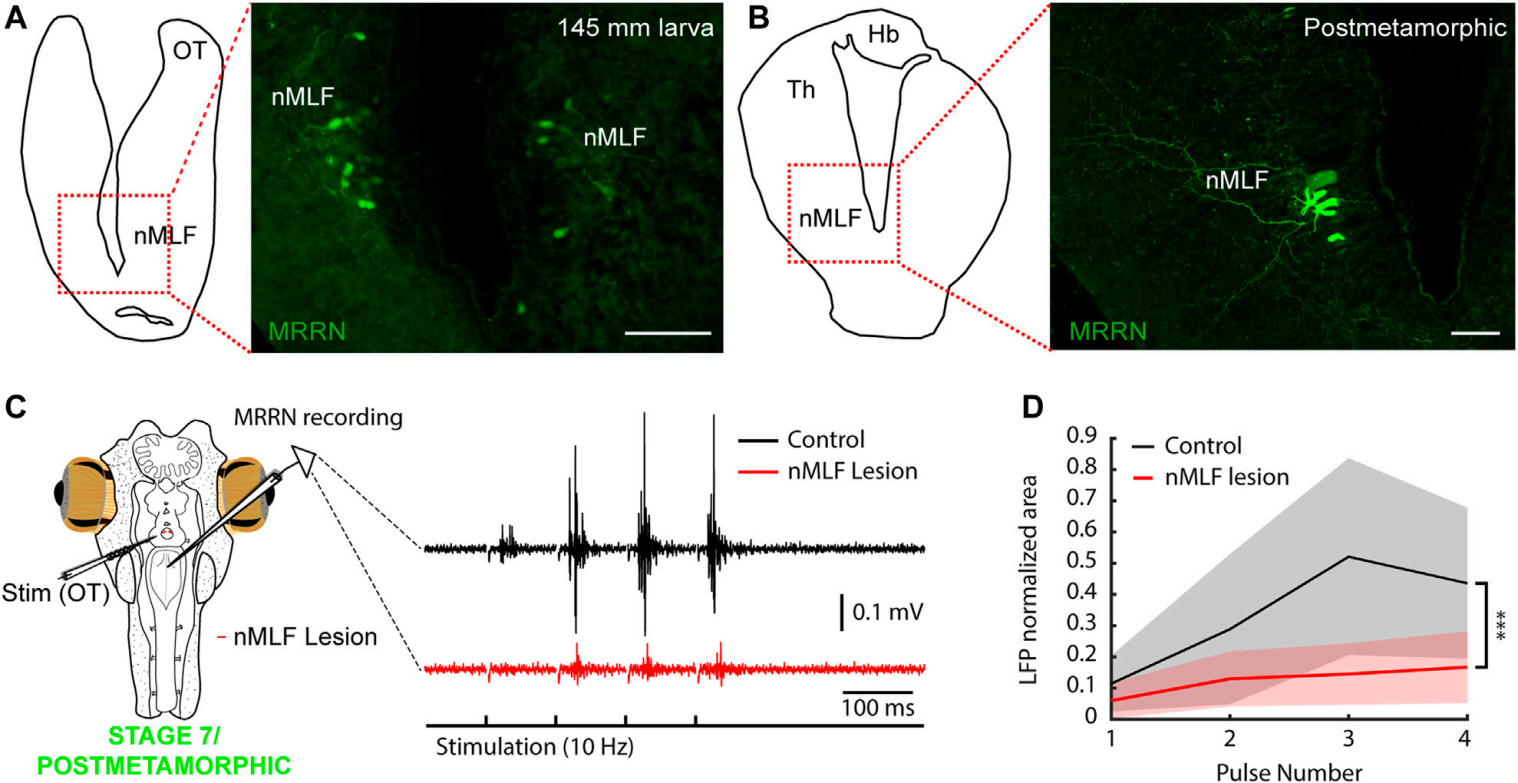
The nMLF mediates tectal responses in larvae and metamorphic lampreys. **(A)** Schematic (left) indicating the location of the photomicrograph (right) showing bilateral retrogradely labeled neurons in the nucleus of the medial longitudinal fasciculus (nMLF) of a 145 mm larva after tracer injection in the middle rhombencephalic reticulospinal nucleus (MRRN). **(B)** Schematic (left) indicating the location of the photomicrograph (right) showing MRRN projecting neurons in the nMLF of a postmetamorphic animal. **(C)** The polysynaptic responses evoked in the MRRN after optic tectum stimulation (OT; black trace) are drastically reduced after lesioning the nMLF in a stage 7/recent postmetamorphic (red trace). Stimulation artifacts were removed for clarity. **(D)** Graph showing the significant reduction in MRRN activity in response to OT stimulation after lesioning the nMLF. Abbreviations: Hb Habenula, Th Dorsal Thalamus. Scale bars = 100 µm in **(A)** and **(B)**.

Although direct tectal projections to the MRRN are not present in larvae of parasitic lampreys, the OT can evoke polysynaptic activity in the MRRN (see above). The main candidate to mediate these motor outputs from the OT is the nMLF. This region sends motor commands to the brainstem and the SC and has been suggested to generate negative phototactic responses (Suzuki and Grillner, 2018). Tracer injections in the MRRN of larvae and early postmetamorphic lampreys showed that the nMLF is the only visual region that projects to the MRRN (Figures 7A,B; N = 16; see also Figure 4A). The other populations projecting to the brainstem were found in the thalamus, likely belonging to the diencephalic locomotor region (El Manira et al., 1997; not shown), and the mesencephalic locomotor region (Grillner and El Manira, 2020; not shown). Thus, we tested whether the nMLF mediates the polysynaptic responses evoked from the OT. For this, we performed electric stimulation of this region in late larvae/metamorphic animals (Figure 7C; 10 Hz, four pulses) and recorded the extracellular activity in the MRRN before (black trace) and after lesioning the nMLF (red trace; N = 4). The evoked responses drastically reduced after the lesion (Figures 7C,D; unpaired *t*-test, *p* = 0.0086; n = 37), indicating that the nMLF is the main structure that mediates the downstream motor responses of the OT.

## 3 Discussion

Lampreys possess a well-developed visual system, with fundamental characteristics found in all vertebrates, whose components and retinofugal pathways develop in a stepwise manner during a long larval period (Suzuki and Grillner, 2018). Here we show that the visuomotor pathways also develop stepwise and that, interestingly, the oculomotor system in lamprey larvae becomes functional long before the development of its visual function, as we show that eye movements are present already in larvae. Previous studies and our tracer injections show that the appearance of VOR corresponds temporally with the establishment of its underlying neural circuits. Interestingly, some degree of eye coordination also appears as soon as VOR emerges. Larval eye movements exhibit slow kinematics compared to the adult ones. However, EMG activity in the extraocular muscles shows that their responses are similar to adults, meaning that the improved eye movement performance is consequence of the eye/orbit development. The number of projections is more evident in larger larvae, which accordingly display a larger range of motion, although larger eye movement amplitudes are likely dependent on an improved muscle function too (Easter and Nicola, 1997). Interestingly, not only VOR but also light evoked eye movements are present in >100 mm larvae. This could be related with the minimum larval size needed to enter metamorphosis, as previously suggested (Pombal et al., 1994).

It is curious to imagine the utility of such an early development of eye movements. Lamprey larvae live buried (Hardisty, 2006) and do not hunt, and their eyes are covered by skin and are not functional image-forming structures (Kleerekoper, 1972). Regarding VOR, it is hard to imagine any behavioral relevance. However, given that both PT/OT and light stimulation evoke eye movements, it is possible that light-evoked ocular movements play a role in phototactic responses. It has been shown that retinal projections to the PT directly target nMLF neurons, suggesting that this pathway can generate negative phototaxis in larvae (Suzuki et al., 2015a; Suzuki et al., 2015b). Our results show a consistent activation of the MRRN via tectal stimulation, mediated via the nMLF. Given the light-evoked MRRN activity in our experiments, the OT can likely also generate phototactic responses through the nMLF. The presence of a basic retinotopy in the larval OT (Cornide-Petronio et al., 2011) could provide a substrate so that motor responses can be evoked with a certain directionality, and therefore light-evoked eye movements may have a behavioral relevance.

**FIGURE 8:**
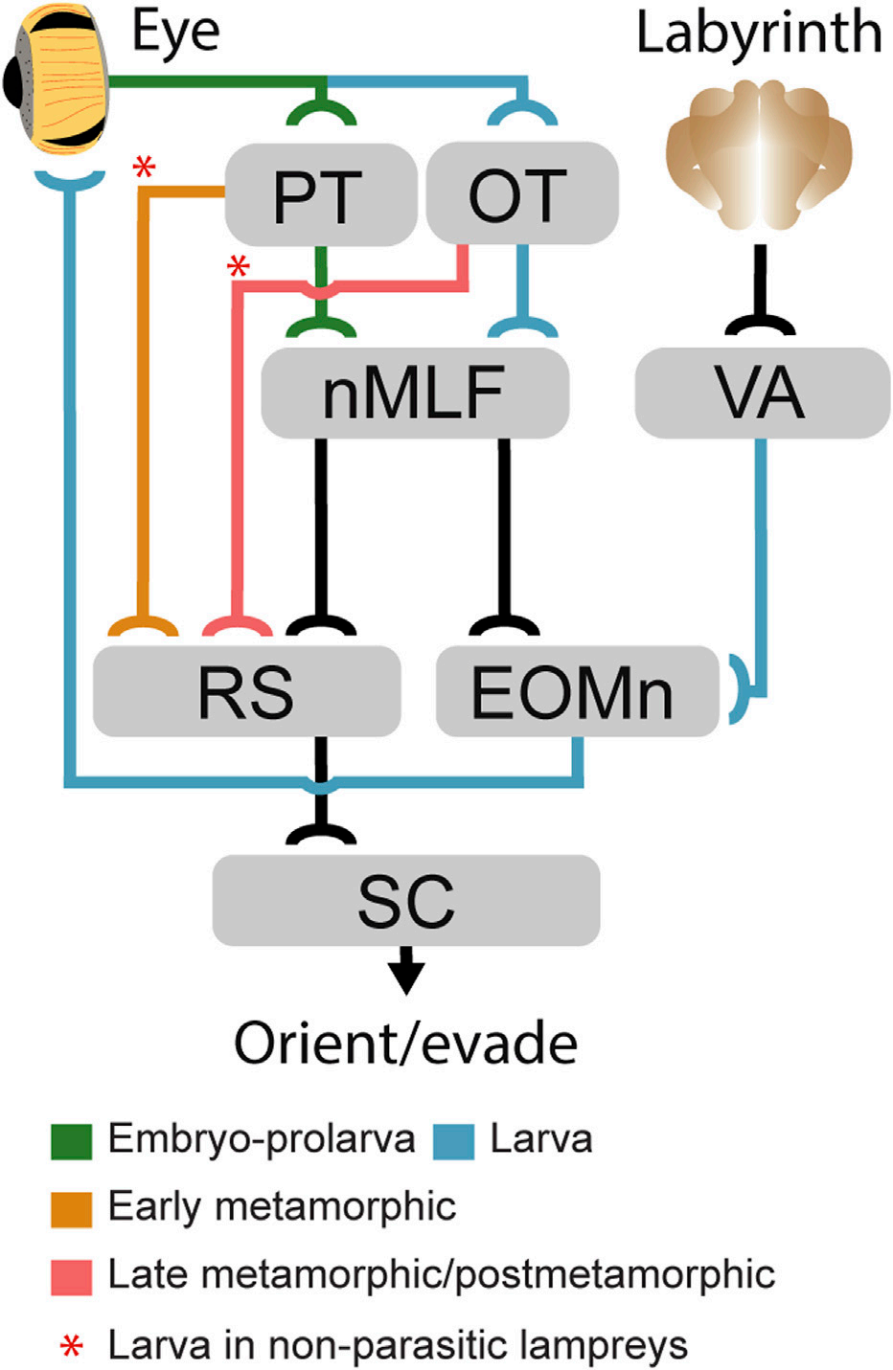
Development of gaze-controlling circuits in lampreys. Schematic showing the appearance stage of the main subcortical pathways mediating gaze control during development. Pathways that develop during embryo-prolarval stage are indicated in green, those stablished in larvae in blue, those that appear at the beginning of metamorphosis in orange, and those that develop only in late metamorphosis are indicated in red. The red asterisks denote pathways that in non-parasitic lampreys start developing in larvae. Abbreviations: EOMn Extraocular Muscles Motor Nuclei; MRRN Middle Rhombencephalic Reticulospinal Nucleus, nMLF Nucleus of the Medial Longitudinal Fasciculus, OT Optic Tectum, PT Pretectum, RS Reticulospinal Neurons, SC Spinal Cord, VA Vestibular A.rea.

It is believed that the first ocular movement that appeared during evolution is the VOR, and that goal-oriented gaze movements evolved from stabilizing responses (Walls, 1962; Land, 2015). We show that the first eye movements during lamprey development are VOR-like followed by phototactic responses in larval stages, and then the OKR followed by goal-oriented responses emerge during metamorphosis, as indicated by the late development of pretectal and tectal motor projections (Figure 8). Remarkably, our results indicate that the pallial control of visuomotor responses appears even later. The development of stabilizing gaze movements first, followed by goal-oriented responses is also observed in other vertebrates (Dayton et al., 1964; Horn et al., 1986; Easter and Nicola, 1997; Huang and Neuhauss, 2008; Branoner et al., 2016; Donovan et al., 2020; Forsthofer and Straka, 2023). For instance, in zebrafish the OKR and the VOR emerge shortly after hatching (73–74 h after fertilization), while spontaneous saccades appear later (81–96 h after fertilization; Easter and Nicola, 1997; Huang and Neuhauss, 2008). The development of the lamprey visual system thus follows the same pattern as in other vertebrates, albeit in a stepwise manner and during a long period spanning several years. It is known that visual experience is necessary for normal development of visuomotor circuits, including the tectal motor maps (du Lac and Knudsen, 1991; Wang et al., 2015), and lamprey development also reflects this. The availability of vestibular and rough light stimuli during the larval period would allow an earlier development of VOR and basic phototactic responses. However, the motor circuits underlying OKR and visually evoked goal-oriented behaviors only develop after the eye can form images and lampreys abandon their buried lifestyle, so that the necessary sensory information is available. Interestingly in parasitic lampreys, which will perform more advanced hunting behaviors, the PT-MRRN connection appears during metamorphosis and the OT-MRRN connection is not found until the last metamorphic stage, and even at this stage the number of tectal neurons that project to the brainstem is very scarce. Thus, it seems that the OT circuits involved in goal-oriented responses are only completed after metamorphosis, when lampreys are exposed to the appropriate visual stimuli in their downstream journey in parasitic lampreys, and as soon as they start swimming in non-parasitic ones. We have not found any evidence of a pallial contribution to gaze responses even in recently transformed animals, suggesting similar sensory requirements and that its development is completed even later, most likely because of its involvement in advanced hunting behaviors. In non-parasitic lampreys, this process is accelerated likely because of their less complex use of the visual system, so that the sensory requirements for its development are also fewer.

Reconstructing the evolutionary history of the visual system is challenging because of the scarce data available in both extant and fossil early vertebrates (Fritzsch, 1991). Thus, understanding its development in lampreys provides valuable insight given their key phylogenetic position (Sugahara et al., 2017). Recent work has suggested that ancient lampreys lacked a filter-feeding stage and larvae had features of modern adult lampreys, including well-developed eyes (Miyashita et al., 2021). While the appearance of new mechanisms was necessary to fulfil the new feeding style (Youson, 1981), the strong similarities of the lamprey visual system and the emergence order of its underlying circuits with that of other vertebrates suggests that its stepwise development is merely a developmental slowing down of the visual system already stablished in ancient lampreys, and that is largely conserved in other vertebrates. This stepwise construction of the visual system offers a unique window to study the role and functioning of the different visuomotor circuits.

## 4 Materials and methods

### 4.1 Animals

Experiments were performed on 51/21 sea lampreys/Nothern Japanese brook lampreys (*P. marinus*/*Lethenteron* sp. N, a cryptic species of *L. reissneri*; Yamazaki and Goto, 1998; Yamazaki et al., 2006), 40/12 larvae, 4/0 metamorphic (transformers), 5/9 postmetamorphic, and 2/0 adult animals. The experimental procedures were approved by the *Xunta de Galicia* under the supervision of the University of Vigo Committee for Animal use in Laboratory in accordance with the current regulations of the European Union (directive 2010/63/EU) and the Spanish regulation (*Real Decreto* 53/2013). A part of this study (using *Lethenteron* sp. N) was performed in accordance with the Regulations on Animal Experimentation at University of Tsukuba. Specific approval is not needed for experimentation on fish under Japanese law, Act on Welfare and Management of Animals. Also, every effort was made to minimize suffering and reduce the number of animals used in the study. The larvae were captured in a tributary of the Miño river (Furnia river: 41°59′33.0 ″N 8°41′33.0 ″W), the transformers were kindly supplied by local fishermen with the permissions from *Xunta de Galicia* and *Comandancia Naval del Miño-Capitanía do Porto de Camiña/Comandancia local da Policía Marítima de Portugal*, whereas adults were obtained from authorized commercial distributors. Larvae and postmetamorphic of *Lethenteron* sp. N were captured from the Kamo River, which flows through the middle of the Shougawa River, Toyama, Japan. Animals were kept in aquaria with an enriched environment and continuously aerated and filtered water.

### 4.2 *Ex-vivo* preparation

To expose the eyes and analyze their movements in response to electric stimulation of the brain, mechanical stimulation of the labyrinth, and light stimulation of the eyes, as well as to perform electrophysiological recordings in different brain regions, an *ex-vivo* preparation was used. For this, specimens were deeply anesthetized with tricaine methanesulfonate dissolved in water (0.1%, MS-222, Sigma). When they stopped responding to tactile stimuli, the head was dissected at the level of the fourth gill and immersed in ice-cooled artificial cerebrospinal fluid (aCSF), with the following composition (in mM): 125 NaCl, 2.5 KCl, 2 CaCl_2_, 1 MgCl_2_, 10 glucose and 25 NaHCO_3_, saturated with 95% (vol/vol) O_2_/5% CO_2_. The skin, muscles and dorsal cartilage of the head were quickly removed to expose the brain. The choroid plexus and pineal gland were also removed. Subsequently, the lateral skin was removed to expose the eyes and otic capsules (containing the vestibular organs). The viscera and all muscles were removed to avoid movement of the preparation. The preparation was allowed to recover for at least 30 min and always kept immersed in cold aCSF during the experiments.

To mechanically stimulate the labyrinth, the cartilage of the otic capsule was cut carefully, so that the labyrinth was not damaged, opening a small window. A thin glass capillary, mounted on a micromanipulator (model M-3333, Narishige), was then gently pushed against the labyrinth membranes for the stimulation. To lesion the nMLF, an incision was made immediately caudal to this nucleus using a microscalpel through the third ventricle, thus cutting its downstream projections to the brainstem.

### 4.3 Eye tracking

To test whether eye movements were evoked in response to electric stimulation of different brain areas, and light or vestibular stimulation, a video camera (AmScope MD35) attached to a stereo microscope (Leica M60) was used to record the eyes. Analysis of eye movements was performed with DeepLabCut (Mathis et al., 2018) a Python-based software package that uses artificial neural networks to estimate poses. To analyze the videos, one to four labels were first placed on each filmed eye in 20 key frames, and then training of the neural network was performed. After evaluating the training, the trained network was used to extract the position of the eyes throughout the video of interest. When several labels were used (in larger eyes), the trajectories of the eye labels were averaged to minimize errors. In some cases, a label was placed on the preparation and used to subtract small movements that might occur in the preparation due to muscle remnants in response to stimulation, or due to camera movements.

### 4.4 Anatomical tract tracing

To study neuronal connections, tracer injections were performed in the AON, PT, retina, MRRN and SC. Injections were performed with glass micropipettes (borosilicate; od = 1.5 mm, id = 1.17 mm; Hilgenberg) with a tip diameter of 10–20 μm or sharpened tungsten pins. The glass micropipettes were attached to a holder connected to a pressurized air supply and this holder was placed on a micromanipulator (model M-3333, Narishige). Using the pressurized air system, the contents of the micropipette (tracer), which consisted of 50–200 nL of 20% (wt/vol) Neurobiotin (Vector Laboratories) in aCSF containing Fast Green (Vector Laboratories), were injected into the brain (in the AON, PT or MRRN). Injections were also performed using dextran amine-tetramethylrhodamine (3 kDa; Molecular Probes). Alternatively, sharpened tungsten pins were mounted on a shaft and fluorescent dextran-amine (tetramethylrhodamine, 3,000 Da, Invitrogen, D3308; Alexa Fluor 488, 10,000 Da, Invitrogen, D22910) was recrystallized onto the tip of the pins (according to Glover, 1995). The crystal was inserted in the brain, retina or SC allowing anterograde or retrograde tracing. After injection, the brains were immersed in aCSF at 4°C for 24–48 h in darkness to allow tracer transport, fixed in 4% formaldehyde and 14% saturated picric acid in 0.1 M saline phosphate buffer (PBS), pH 7.4, for 12–24 h, and cryoprotected in 20% (wt/vol) sucrose in PBS for 3–12 h. Subsequently, they were embedded in OCT compound (Tissue-Tek, Sakura) and transverse sections of 30 μm thickness were cut in a cryostat (Leica cm 1950) and collected on gelatinized slides. Before sectioning, some samples were dehydrated with a series of methanol in PBS (25%, 50%, 75%, 100%, and again 100% for 15 min each at room temperature) and clarified with BABB (1:2 mixture of benzyl alcohol and benzyl benzoate, for 15 min at room temperature). After the whole mount observation of the clarified sample with a confocal laser microscope (LSM 510, Zeiss, Göttingen, Germany), the samples were rinsed with 100% methanol (for 15 min, two times, at room temperature), rehydrated with the series of methanol in PBS (75%, 50%, and 25%), and then rinsed with pure PBS (for 15 min, two times, at room temperature) for the subsequent sectioning procedure.

To detect the Neurobiotin tracer, sections were incubated in Cy2-conjugated streptavidin (Jackson ImmunoResearch) 1:1,000 in blocking solution (1% bovine serum albumin, 10% sheep serum, 0.1% sodium azide and 0.3% Triton X-100 in PBS). A Nissl stain (Molecular Probes) was also added (1:500). Incubation was performed in a dark humid chamber for 2 h, and after three 10 min washes with PBS they were mounted in glycerol (Panreac).

### 4.5 Electrophysiology

Extracellular recordings were performed in reticulospinal neurons in response to electrical stimulation of the AON, PT and OT, and to mechanical stimulation of the labyrinth using custom tungsten microelectrodes (∼1–5 MΩ). EMG recordings were also performed in the extraocular muscles. The recording electrodes were connected to an amplifier (AC differential amplifier, model 1,700, AM systems) and the signal obtained digitized at 20 kHz using pClamp 10.4. The electrodes were operated using a micromanipulator (model M-3333, Narishige) that allowed their stable and precise positioning.

For electrical stimulation, borosilicate pipettes filled with aCSF were used, connected to a stimulus isolation unit (MI401). The stimulation intensity was set according to the threshold strength (usually 10–100 μA) necessary to evoke neuronal activity. For mechanical stimulation, a small window was made into the otic capsule, from which the vestibular system was directly stimulated by pressing the labyrinth with the tip of a pipette moved using a micromanipulator (model M-3333, Narishige).

### 4.6 Image analysis

Photomicrographs were taken with a digital camera (Nikon DS-Ri2) mounted on a Nikon ECLIPSE Ni-E fluorescence microscope. Illustrations were made using Adobe Illustrator CC 2019, GIMP 2.1 (GNU image manipulator program), and Paint 3D. Images were only adjusted for brightness and contrast. The samples clarified with BABB were examined using a confocal laser micro-scope (LSM 510, Zeiss, Goettingen, Germany).

### 4.7 Quantification and statistical analysis

For both electrophysiological and eye tracking data, analyses were performed using custom written functions in Matlab. For video recordings, eye positions were extracted with DeepLabCut (Mathis et al., 2018; see above). When only one eye was recorded frontally, to calculate the amplitude of the eye movement, the recorded eyes were extracted and measured in millimeters (mm) after the experiments, and the diameter of the eye was compared to its image so that a reliable conversion index from pixels to mm could be established. The video recorded eye movement amplitudes were consequently translated into mm. Using the diameter of the eye in the Z-axis (distance from the optic nerve entrance to the cornea), its angular displacement could be calculated using trigonometric functions. To analyze the degree of eye coordination, a linear regression analysis was performed by calculating Pearson’s coherence coefficient.

To compare the signals evoked after each of the electric stimulation pulses, the integral under the curves were calculated after full rectification of the signals using trapezoidal numerical integration (‘trapz’ function) and the resulted values were normalized to the first pulse. To investigate the polysynaptic nature of the extracellular responses recorded, onset latencies were extracted by calculating the time from stimulus application to the first spike of the neuronal response. To compare latency times among different developmental stages, and the differences in MRRN activity in response to OT stimulation before and after nMLF lesioning, unpaired *t*-tests were used. The number of animals (N) and the number of experiments performed (n) are indicated where applicable. The degree of significance is indicated as follows: *p < 0.05, **p < 0.01, ***p < 0.001.

## Data Availability

The raw data supporting the conclusion of this article will be made available by the authors, without undue reservation.
