# Supplementary material for: Unravelling the functional development of vertebrate pathways controlling gaze
Source: Front Cell Dev Biol. 2023 Oct 26;11:1298486. doi: 10.3389/fcell.2023.1298486 (PMC10640995; doi:10.3389/fcell.2023.1298486)
Supplement: Supplementary file 2 [file DataSheet1.PDF]

Supplementary Material

Unravelling the functional development of vertebrate pathways controlling gaze

Marta Barandela, Carmen Núñez-González, Daichi G. Suzuki, Cecilia Jiménez-López, Manuel A. Pombal, Juan Pérez-Fernández*

*** Correspondence:** Corresponding Author: jperezf@uvigo.es

Supplementary Figures

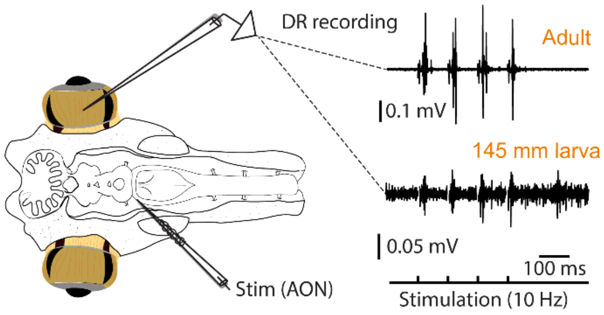

Supplementary Figure 1. Extraocular muscles show similar activity in both larvae and adult lampreys. EMG activity in the dorsal rectus (DR) in of an adult lamprey (top trace) and a 145 mm larva (bottom trace) in response to a four pulses stimulation (10 Hz) of the anterior octavomotor nucleus (AON).

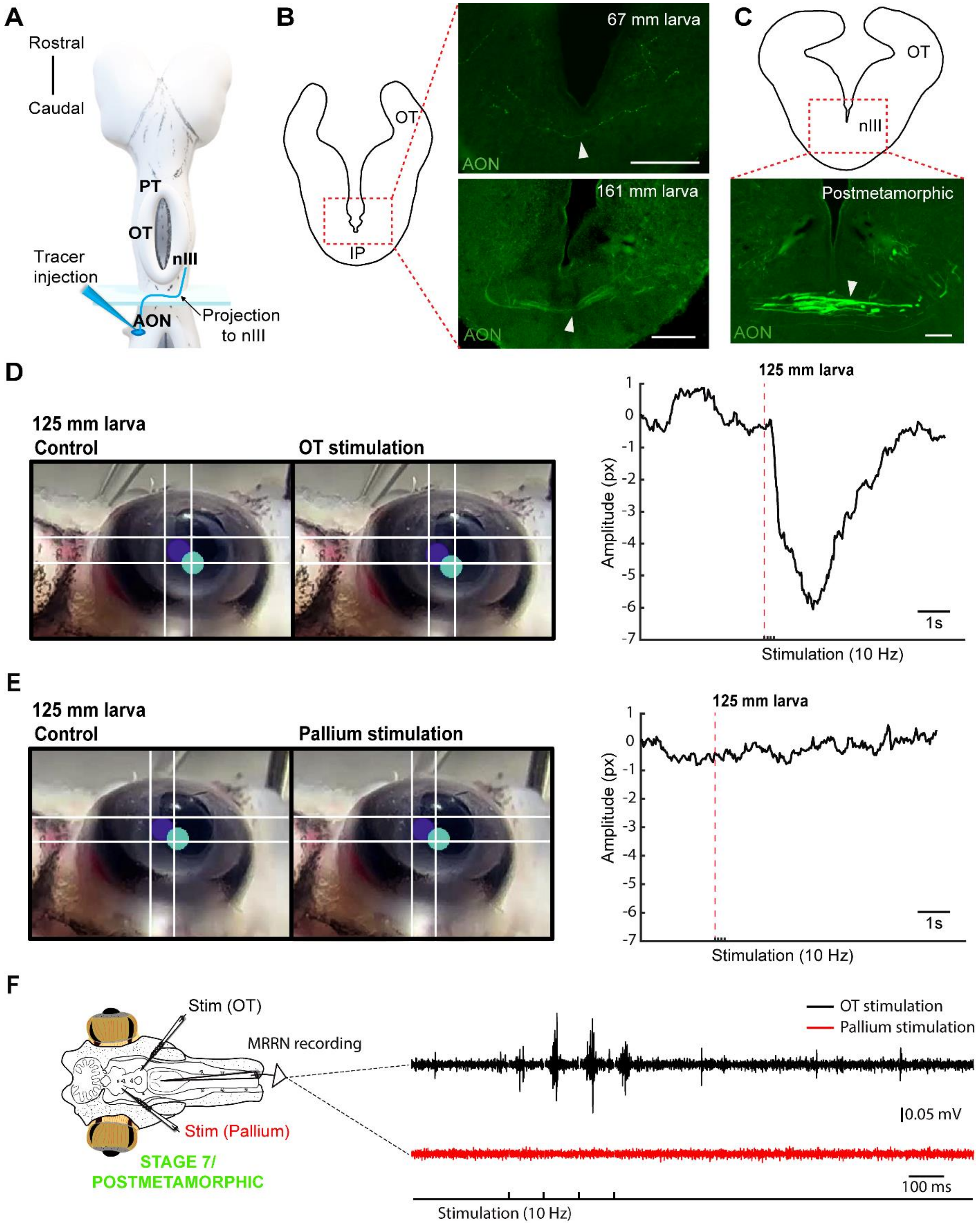

Supplementary Figure 2. AON fibers crossing to the oculomotor nucleus. Schematic dorsal view of the lamprey larva brain indicating the tracer injection site in the anterior octavomotor nucleus (AON) from where the projections (blue line) that reach the oculomotor nucleus (nIII) originate. The rectangle indicates the location of the photomicrographs in (B) and (C). (B) Anterogradely labeled

fibers crossing at the level of the interpeduncular nucleus (IP) in a 67 mm larva (top) and a 161 mm larva (bottom). (C) Fibers crossing at the same location than (B) in a postmetamorphic animal. (D) Right: Trace showing the position of the eye in response to a four pulses 10 Hz stimulation of the optic tectum (OT) in a 125 mm larva. Left: Frames showing the position of the eye before (left) and after (right) OT stimulation. (E) In the same larva, no eye movements are evoked after pallial stimulation. Right: Trace showing the position of the eye in response to a four pulses 10 Hz stimulation of the visual region in pallium in the same 125 mm larva. Left: Frames showing the position of the eye before (left) and after (right) pallial stimulation. (F) Stimulation of the visual area in pallium does not result in neuronal activity in the middle rhombencephalic reticulospinal nucleus (MRRN; red trace) of a stage 7/postmetamorphic animal, although activity is recorded in this region after stimulation of the OT (black trace). Scale bar = 100 μm (B) and (C).

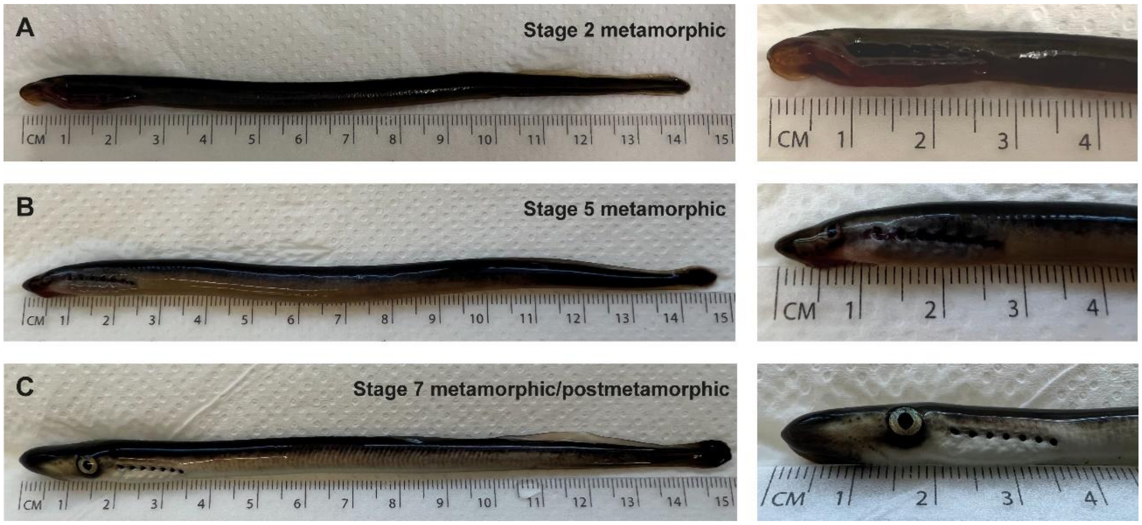

Supplementary Figure 3. Representative metamorphic animals. (A-C) Photographs of representative metamorphic animals used in this study. A stage 2 metamorphic animal is shown in (A), a stage 5 in (B), and a stage7/postmetamorphic animal is shown in (C). A more detailed view of the head is shown in the right. The stage classification is based on Youson and Potter (1979).
